# A suspected case of carbimazole-associated torsades de pointes

**DOI:** 10.4103/0253-7613.62406

**Published:** 2010-02

**Authors:** Chiranjib Bagchi, Dhurjati Prasad Sinha, Santanu Kumar Tripathi

**Affiliations:** Department of Pharmacology, Burdwan Medical College, Burdwan, West Bengal 713104, India; 1 Department of Cardiology, Burdwan Medical College, Burdwan, West Bengal 713104, India

**Keywords:** Carbimazole, QT prolongation, torsades de pointes

## Abstract

Torsades de pointes (TdP) or polymorphic ventricular tachycardia owing to drug-induced QT prolongation is a common cause of withdrawal of marketed drugs and has caused increasing concern in the recent past. Carbimazole, the common antithyroid drug, is not a very well-known offender to cause QT prolongation and TdP. Only a few cases of carbimazole-induced TdP have been reported so far. We report a 53-year-old woman who was on tab. carbimazole (10 mg) twice daily for one month and who presented with respiratory distress, palpitation and syncope attack. Her surface electrocardiogram (ECG) was showing the evidence of TdP and subsequently hypokalemia was also detected. She received conservative management including potassium supplementation. However, QT prolongation persisted even after normalization of serum potassium level. Carbimazole was withdrawn and the patient was discharged as she remained stable and symptom free. This study highlights a possible association between carbimazole and TdP.

## Introduction

Torsades de pointes refers to a ventricular tachycardia (VT) characterized in surface ECG by QRS complexes of changing amplitude that appear to twist around the isoelectric line and occur at a rate of 200–250/min. Therefore, it is a form of polymorphic VT which is characterized by prolonged ventricular repolarization with QT intervals exceeding 500 ms.[1] The VT that is similar in morphology to torsades but occurs without QT prolongation, should be classified as polymorphic VT, but not as torsades. It is often reported that women are at greater risk of TdP than men, perhaps because of longer QT interval.[1]

QT interval is measured from the beginning of the QRS complex to the end of the T wave in the surface ECG. Corrected QT interval (QTc) is the QT interval which is adjusted for the heart rate. The most commonly used formulae for QTc calculation are Fridericia's cube root formula (QTc = QT/RR^1/3^) and Bazett's square root formula (QTc = QT/RR^1/2^). Bazett's formula is more popular than Fridericia's formula, but Fridericia's correction is preferred because it is more accurate at the extremes of physiological heart rate.[2] QT interval is found to be prolonged when QTc is ≥450 ms (woman) and ≥430 ms (man).[3]

Among the various causes of TdP, drug-induced QT prolongation appears to be the most common. Cardiovascular drugs such as class I antiarrhythmics are critical in this respect and are contraindicated in the treatment of TdP. However, it is more alarming when TdP occurs with other frequently used drugs such as antibiotics, antihistaminics, antipsychotics, etc.[2] and though rarely, with commonly used antithyroid drugs like carbimazole.[4,5]

Carbimazole is an orally administered thionamide type of antithyroid drug. This is a prodrug and is converted to methimazole after absorption. Carbimazole is a synthesis inhibitor, affects iodination of thyroglobulin and also inhibits the coupling reaction. Here we report a case of prolonged QT syndrome with polymorphic VT, i.e. TdP which developed after 1 month of therapy with carbimazole.

## Case Report

**Figure 1:**
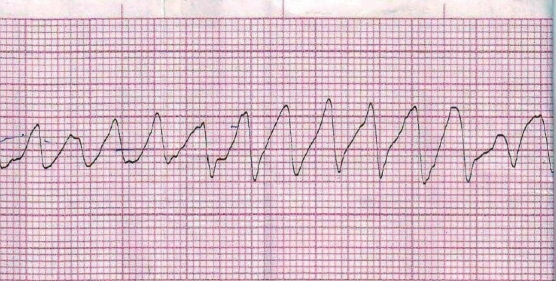
Lead II of ECG showing TdP (recorded at 25 mm/s and 1 mV = 10 mm calibration).

**Figure 2:**
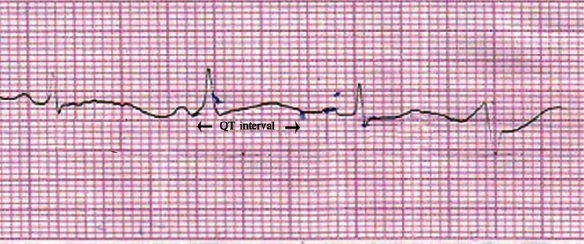
Lead II of ECG showing QT prolongation (QTc = QT/RR^1/2^ = 550 ms)

A 53-year-old woman was brought to the emergency department of Burdwan Medical College and Hospital in the night on November 18, 2008 with respiratory distress, palpitation and blood tinged frothing from mouth. Although she was conscious initially, she developed a syncope attack after few minutes. Her ECG revealed the evidence of polymorphic VT i.e. TdP which was self-terminating [Figure 1]. Applying both Bazett's formula and Fridericia's formula, the QTc intervals appeared to be prolonged with values of 550 ms and 511 ms respectively [Figure 2]. Her medication history revealed that she had been receiving tab. ramipril 1.25 mg once daily and tab. carbimazole 10 mg twice daily since last 1 month. In the emergency room (ER), inj. frusemide, inj. Deriphyllin (combination of etophylline and theophylline) and inj. dexamethasone, each 1 amp intravenously stat, were administered. She was then transferred to intensive coronary care unit. Inj. frusemide, started in the ER, was continued as 1 amp. intravenously twice daily. Investigations on the following day indicated the presence of hypokalemia and hyponatremia with serum potassium and sodium levels at 2.6 mmol/L (normal 3.5–5 mmol/L) and 133 mmol/L (normal 135–145 mmol/L), respectively. Inj. frusemide was then replaced with combination of tab. frusemide plus spironolactone. Normal saline (NS) infusion started with inj. potassium chloride (20 meq) added to each bottle of NS. Serum sodium and potassium levels were normalized within 24 hours. Her thyroid profile showed euthyroid state with normal serum T_3_ 148.2 ng/dL (normal 70–190 ng/dL) and T_4_ 11.06 *μ*g/dL (normal 5–12 *μ*g/dL) with low serum TSH 0.13 *μ*U/mL (normal 0.4–0.5 *μ*U/mL). QT prolongation, however, persisted even after normalization of serum potassium level. The patient was treated conservatively and tab. carbimazole was withdrawn. She remained stable and symptom-free and was discharged with instruction to attend the outpatient department regularly.

The patient was admitted in the same hospital 3 months ago on August 01, 2008 for ventricular ectopics, but there was no evidence of QT prolongation. Subsequently during follow-up visits, hyperthyroidism was detected and tab. carbimazole was administered (10 mg twice daily) since 1 month.

## Discussion

Considering the causes of TdP, the probabilities in this case were analyzed. Hypokalemia is well known to cause QT prolongation and TdP, but in this study QT prolongation persisted even after normalization of serum potassium level. As QT prolongation persisted even after termination of TdP and normalization of serum potassium level, TdP could have been precipitated by hypokalemia interacting with carbimazole.

Frusemide is known to cause hypokalemia. However, in this case TdP manifested before the administration of frusemide. Therefore, an association between frusemide and TdP does not exist. At best frusemide-induced hypokalemia if any, might have aggravated the condition. Frusemide was given initially as the patient had the symptoms of pulmonary edema possibly caused by the impairment of left ventricular ejection due to VT.

There may be a probability of hereditary long QT syndrome. However, during her previous hospital admission and subsequent follow-up visits, there was no evidence of prolonged QT interval. Although carbimazole is not well known to prolong QT interval, there are few reports of its association with TdP.[4,5] In a patient of thyrotoxicosis with bradycardia owing to atrioventricular conduction block, occurrence of hypothyroidism owing to overtreatment with carbimazole was proposed to initiate the TdP.[4] In the present study, the thyroid profile was normal during her hospital stay while she was on carbimazole.

A suspected drug interaction between carbimazole and erythromycin taken orally thereby increasing the serum concentration of the later and producing TdP has been reported. Carbimazole has recently been known as a hepatic microsomal enzyme inhibitor.[5] In the present case, the only concomitant medication the patient was receiving was tab. ramipril (1.25 mg) once daily which is not known to either prolong QT or cause TdP. The association between carbimazole and TdP was evaluated using both Naranjo's Causality Assessment Scale[6] and World Health Organisation (WHO) Uppsala Monitoring Centre (UMC) Causality Assessment Criteria.[7] Naranjo's scale revealed a score of 3, signifying a possible association. The WHO-UMC scale also indicated a possible association. In conclusion, this case report suggests a possible association between carbimazole therapy and occurrence of TdP. Further studies are warranted in order to find the definite cause of carbimazole-associated TdP.
